# Primary Parotid Tumor Thrombosis: Immunohistologic Features and Awareness of Metastatic Potential

**DOI:** 10.7759/cureus.16174

**Published:** 2021-07-04

**Authors:** Antonio Dekhou, Rafey Rehman, Jacob S Parzen, Thomas J Quinn, Ping L Zhang, Matthew Rontal, Samir Noujaim, Martin Tapia, Rohan Deraniyagala

**Affiliations:** 1 Department of Radiation Oncology, Oakland University William Beaumont School of Medicine, Rochester, USA; 2 Department of Radiation Oncology, Beaumont Health, Royal Oak, USA; 3 Department of Pathology, Beaumont Health, Royal Oak, USA; 4 Department of Otolaryngology, Beaumont Health, Royal Oak, USA; 5 Department of Radiology, Beaumont Health, Royal Oak, USA; 6 Department of Hematology Oncology, Beaumont Health, Royal Oak, USA

**Keywords:** myoepithelial carcinoma, tumor thrombosis, sox10, myosin, immunohistochemical staining

## Abstract

Tumor thrombosis is a poor prognostic feature and an exceptionally rare occurrence in salivary gland malignancies. We present a case of primary parotid myoepithelial carcinoma (MC) with tumor thrombosis in the external jugular vein (EJV). An 82-year-old man presented with a right-sided facial mass. MRI with and without gadolinium demonstrated a mass of the right parotid gland with a filling defect of the right EJV. The patient underwent right parotidectomy and selective neck dissection. Tumor thrombosis was found intraoperatively within the EJV. Final pathology demonstrated a poorly differentiated MC. Adjuvant radiation therapy without concurrent systemic therapy was administered. Three months later, restaging positron emission tomography (PET) with CT revealed numerous bilateral pulmonary nodules with biopsy, demonstrating poorly differentiated MC without locoregional relapse. Given that primary parotid tumor thrombosis is associated with a poor prognosis, the use of early systemic therapy should be investigated.

## Introduction

Myoepithelial carcinoma (MC), also known as malignant myoepithelioma, is a rare salivary gland malignancy that accounts for fewer than 1% of salivary gland tumors, most commonly arising in the parotid gland [1,2]. MC tends to occur in middle-aged individuals, with a median age of diagnosis ranging from 51 to 61 years, with no clear gender predominance [3,4]. Due to its rarity, the clinical presentation, management, and complications of MC of the parotid gland have not been well-documented. Tumor thrombosis due to a primary MC of the parotid gland tumor is particularly rare, and to the best of our knowledge, a primary parotid MC with tumor thrombosis has not been previously reported [5]. Here, we present an unusual case of MC of the right parotid gland with tumor thrombosis of the external jugular vein (EJV) with rapid metastatic dissemination in a manner consistent with hematogenous spread following locoregional therapy.

## Case presentation

An 82-year-old man with a past medical history of hypertension and a remote history of cigarette smoking presented to his otolaryngologist with a primary concern of a palpable mass on the right side of his neck. CT of the neck without intravenous (IV) contrast demonstrated no discernible abnormalities within the right parotid gland, though the right retromandibular vein was slightly larger than the left. A follow-up ultrasound demonstrated a focal mass-like abnormality in the deep parotid gland extending around the angle of the right mandible, measuring 2.6 cm by 1.8 cm. There was also a re-demonstration of the prominent retromandibular vein. Repeat ultrasound demonstrated no discernible mass, and the abnormality within the right parotid gland was thought to represent a thrombosed retromandibular facial vein. Magnetic resonance angiography (MRA) of the brain with and without gadolinium revealed an ill-defined region of enhancement measuring 3.0 cm by 1.7 cm in the deep right parotid gland extending medially adjacent to the carotid space and right masticator space. Ultrasound-guided fine-needle aspiration of the parotid mass demonstrated a myoepithelial neoplasm. Additional imaging studies, surgical resection of the parotid tumor, and pathology evaluation were performed. 

**Figure 1:**
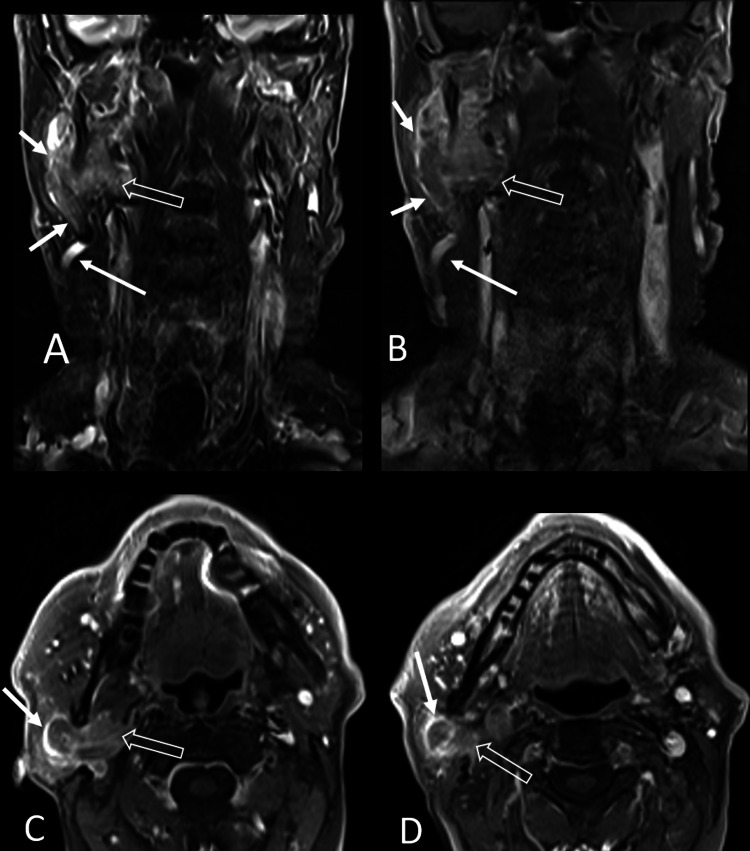
MRI of the neck showing right parotid gland myoepithelial carcinoma. (A) Coronal T2 WI fat-suppressed. (B) Coronal T1 fat saturation, contrast enhancement. (C-D) Axial T1 WI fat saturation, contrast enhancement, higher and lower plane. A lobulated, well-defined mass is noted affecting the superficial and deep lobes of the right parotid gland with high signal on T2WI and strong post gadolinium enhancement (open arrows). Adjacent and lateral to the mass, there is a vertically oriented tubular structure corresponding to a dilated retromandibular vein expanded by intraluminal tumor thrombus (short arrows). Caudally, this vein is in continuity with the external jugular vein (long arrows). WI: Weighted imaging.

MRI of the neck with and without gadolinium demonstrated a 3.5 cm by 2.8 cm by 5.2 cm lobulated mass within the deep and superficial portions of the right parotid gland. Interestingly, there was an abrupt cut-off of the asymmetrically enlarged right EJV and compression of the right internal jugular vein (IJV) at the level of the mass, which was concerning for segmental venous occlusion (Figure 1). Positron emission tomography with CT (PET-CT) demonstrated the right parotid mass to have a standard uptake value of 10.1. There were no additional fluorodeoxyglucose (FDG)-avid foci to suggest regional or metastatic spread or synchronous cancers.

The patient underwent a right parotidectomy with facial nerve monitoring and a right selective neck dissection of levels II-IV. Intraoperatively, a large, dilated vein was appreciated grossly within the mass that appeared to contain the tumor. Final pathology demonstrated a 5.5-cm poorly differentiated MC involving the parotid gland and surrounding soft tissue with perineural invasion and extensive angiolymphatic invasion. There was also gross invasion into vessels forming nodules and tumor thrombus. Tumor cells were present 1 mm from the superior margin and <1 mm from the medial margin. Nine lymph nodes were dissected from the right neck (levels 2, 3, and 4), all of which were negative for evidence of malignancy.

**Figure 2:**
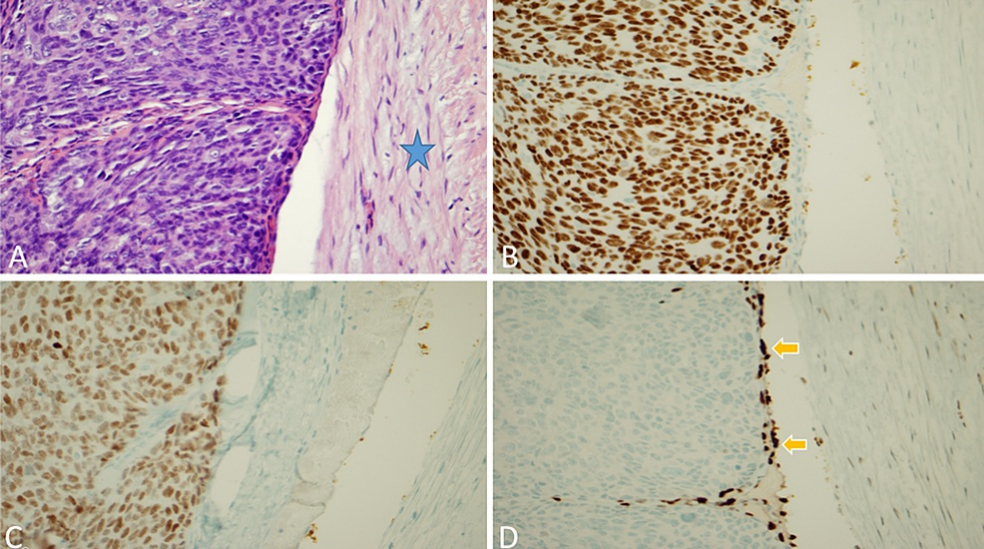
Histologic evaluation and staining of myoepithelial carcinoma. (A) Basaloid features of poorly differentiated carcinoma with polygonal cellular configuration (left side) within a vascular structure (blue star). (B) The tumor cells stained positively for nuclear p40; thus, the differential diagnosis included squamous cell carcinoma versus myoepithelial carcinoma. (C) The tumor cells stained positively for nuclear SOX10 (and focally positive for smooth muscle myosin [not shown]), suggesting a myoepithelial carcinoma. (D) The vascular invasion was confirmed by positive nuclear ERG staining for endothelial cells (two orange arrows) compatible with the clinical impression of venous tumor thrombi. ERG: Erythroblast transformation-specific-related gene; SOX10: Sex-determining region Y-box transcription factor 10.

The tumor was positive for cytokeratin 5/6 (CK5/6), p16, p40, sex-determining region Y-box transcription factor 10 (SOX10), smooth muscle actin, myosin, and synaptophysin, but negative for insulinoma-associated protein 1 (INSM1), nuclear carcinoma of the testis (NUT), S100, erythroblast transformation-specific-related gene (ERG), and uroplakin. However, the screen for human papillomavirus was negative on subsequent ribonucleic acid in situ hybridization study. Tumor positivity for nuclear p40 encompassed the differential diagnoses of squamous cell carcinoma versus MC of the parotid gland. However, subsequent positivity for nuclear SOX10 and focal positive staining for myosin favored the diagnosis of MC [6]. Figure 2 depicts the basaloid features of this poorly differentiated carcinoma that immunohistochemically stained positively for nuclear p40 and SOX10, consistent with MC of the parotid gland and the positive nuclear ERG staining for endothelial cells, confirming vascular invasion in our patient.

The patient subsequently underwent adjuvant radiation therapy to the right parotid bed, base of the skull, and the right side of levels II through IV. A volumetric arc modulated therapy plan with 6 MV photons was used to deliver a dose of 70 Gy in 35 daily fractions prescribed as a minimal peripheral dose to the planning target volume. No concurrent systemic chemotherapy was administered.

He tolerated treatment well with minimal dysgeusia and xerostomia. He had a restaging PET-CT three months later, which demonstrated innumerable bilateral pulmonary nodules suspicious for pulmonary metastasis. There was no local or regional relapse. A chest CT without IV contrast confirmed numerous bilateral pulmonary nodules measuring up to 1 cm. CT-guided biopsy of a left lower lobe nodule was completed, with pathology demonstrating poorly differentiated MC consistent with metastasis from the patient’s previous primary lesion. He was started on pembrolizumab and was alive at the time of this writing.

## Discussion

MC of the parotid gland is a rare salivary gland malignancy, especially when presenting with tumor thrombosis in adjacent vessels [3,4]. Due to the rare occurrence of MC of the parotid gland, its clinical evaluation and management have not been well-documented in the literature. 

To date, there have been four studies reporting tumor thrombosis associated with a parotid gland tumor [5, 7-9]. However, to the best of our knowledge, this is the first reported case of tumor thrombosis of the EJV with subsequent distant pulmonary metastasis. Tumor thrombus, also known as intravascular tumor extension, is defined as tumor extension into a vessel [10]. Tumor thrombosis can occur secondary to malignancy via direct compression of the adjacent vasculature leading to venous stasis or via direct tumor extension within the vein [5]. The differentiation of the mechanisms of tumor thrombosis is critical to determine treatment outcomes, as the degree of surgical resection and amount of radiation necessary for treatment differ depending on the exact etiology for certain tumors [5,10]. Head and neck cancers (HNC) possess biological factors that are considered high risk for tumor thrombus formation, which include modified thrombosis/fibrinolysis mechanisms, platelet activation, and excessive platelet degranulation and release of vascular endothelial growth factor (VEGF), epidermal growth factor (EGF), and platelet-derived growth factor (PDGF) [11]. Of the variety of HNC, follicular thyroid carcinoma is more commonly implicated in tumor thrombus formation given its propensity for hematogenous spread, occurring at an estimated rate of 1.3% [12]. However, the rate of tumor thrombosis in other HNC such as squamous cell carcinomas or salivary gland carcinomas is not well-documented given the rare occurrence in these cases [13,14].

Typically, MC is known to spread via the lymphatic system rather than the hematogenous route, and therefore, regional control is attempted through neck dissection. Several mechanisms of metastatic spread associated with tumor thrombus through the venous system have been proposed. Direct contiguous extension of the tumor thrombus into draining veins can result in seeding the primary tumor, resulting in hematogenous dissemination of the tumor along with the venous drainage system [15]. Given the complex venous drainage system, the lungs are a common site of metastasis for breast tumors, melanomas, sarcomas, and lower rectal tumors [15]. Importantly, initial metastasis to the lungs can further disseminate tumor cells into the systemic circulation, thereby worsening patient outcomes [15]. 

Radiological investigations for tumor thrombus in those with malignancies are crucial, as its presence may act as an independent risk factor for metastasis [15]. The current literature emphasizes the importance of detecting tumor thrombosis in various malignancies, especially in renal cell carcinoma and hepatocellular carcinoma [16-18]. However, tumor thrombus in the context of other malignancies should be explored further, especially in salivary gland malignancies.

Given the intimate relationship of the parotid gland with the retromandibular vein, EJV, and IJV, radiologic features of tumor thrombus with a concurrent parotid gland malignancy should raise concern for possible metastasis. MRI is considered the most appropriate method for evaluating parotid gland tumors, given its high-contrast resolution for soft tissues and capability to assess perineural and vascular invasion thoroughly. Both CT and MRI can also be beneficial in detecting tumor thrombus and characterizing the extent of tumor extension [10]. In our case, the patient did not present with any particular symptom, and an enlarged vein was first noted incidentally on MRA of the brain with gadolinium. Afterward, an MRI of the neck with gadolinium revealed an asymmetrically enlarged right EJV and attenuated blood flow in the right IJV at the level of the mass. 

In our case, the patient presented with features associated with tumor thrombus, which should increase suspicion for possible metastatic spread. At initial presentation, the patient had slight enlargement of the right retromandibular vein compared to the left, with subsequent imaging displaying re-demonstration of the right retromandibular vein. However, the patient underwent a right parotidectomy and a selective neck dissection with subsequent radiation to the parotid bed and skull base. Concurrent systemic therapy was not recommended given the low risk of metastasis seen in MC of the parotid gland and the unclear role of concurrent systemic therapy in the treatment of salivary gland malignancies [16,19]. The Radiation Therapy Oncology Group (RTOG) 1008 is an open randomized trial investigating the use of chemoradiation compared to radiation therapy alone in the treatment of salivary gland carcinomas [20]. Given the possibility of early metastasis secondary to tumor thrombosis, the use of systemic chemotherapy in the treatment of MC of the parotid gland should be investigated. The development of subsequent bilateral pulmonary metastases secondary to primary MC of the parotid gland may have been prevented with the use of early systemic chemotherapy.

## Conclusions

Tumor thrombosis is a well-documented negative prognostic factor in various malignancies. Early detection and diagnosis of MC of the parotid gland are crucial, especially in those with a concurrent presentation of tumor thrombosis as documented in our patient. Primary parotid tumor thrombosis is rare and carries a poor prognosis. The use of early systemic chemotherapy should be investigated.
